# Ultrafast Momentum-Resolved Hot Electron Dynamics in the Two-Dimensional Topological Insulator Bismuthene

**DOI:** 10.1021/acs.nanolett.2c01462

**Published:** 2022-06-16

**Authors:** Julian Maklar, Raúl Stühler, Maciej Dendzik, Tommaso Pincelli, Shuo Dong, Samuel Beaulieu, Alexander Neef, Gang Li, Martin Wolf, Ralph Ernstorfer, Ralph Claessen, Laurenz Rettig

**Affiliations:** † Fritz-Haber-Institut der Max-Planck-Gesellschaft, Faradayweg 4-6, D-14195 Berlin, Germany; ‡ Physikalisches Institut and Würzburg-Dresden Cluster of Excellence ct.qmat, University of Würzburg, D-97070 Würzburg, Germany; ¶ School of Physical Science and Technology, ShanghaiTech University, Shanghai 200031, China; § Institut für Optik und Atomare Physik, Technische Universität Berlin, Straße des 17. Juni 135, 10623 Berlin, Germany

**Keywords:** Topological insulators, quantum spin Hall effect, bismuthene, time- and angle-resolved photoemission spectroscopy, trARPES, ultrafast carrier dynamics

## Abstract

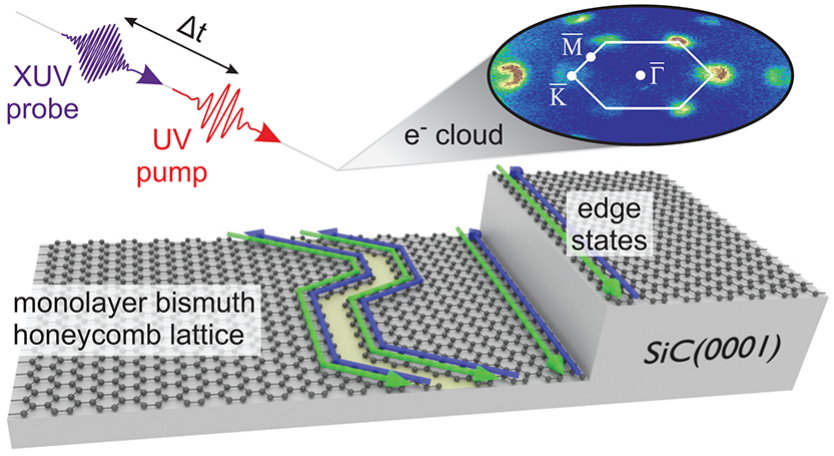

Two-dimensional quantum spin Hall (QSH) insulators are a promising material class for spintronic applications based on topologically protected spin currents in their edges. Yet, they have not lived up to their technological potential, as experimental realizations are scarce and limited to cryogenic temperatures. These constraints have also severely restricted characterization of their dynamical properties. Here, we report on the electron dynamics of the novel room-temperature QSH candidate bismuthene after photoexcitation using time- and angle-resolved photoemission spectroscopy. We map the transiently occupied conduction band and track the full relaxation pathway of hot photocarriers. Intriguingly, we observe photocarrier lifetimes much shorter than those in conventional semiconductors. This is ascribed to the presence of topological in-gap states already established by local probes. Indeed, we find spectral signatures consistent with these earlier findings. Demonstration of the large band gap and the view into photoelectron dynamics mark a critical step toward optical control of QSH functionalities.

A promising platform for spintronic devices are two-dimensional (2D) topological insulators (TIs).^1^ Based on the quantum spin Hall (QSH) effect, 2D TIs feature an insulating band structure in their interior (here referred to as 2D bulk) surrounded by metallic states at their edges. These helical edge states (ESs) are characterized by spin-momentum locking, allowing for spin currents with opposite polarization for forward- and backward-moving electrons. As they are topologically protected by time-reversal symmetry against single-particle backscattering, they also enable dissipationless transport.^2,3^ However, any practical application based on helical ESs requires a large bulk band gap preventing interference with thermally excited bulk carriers at room temperature. Thus, in addition to a thorough characterization of the ES properties, also mapping out the bulk valence and conduction bands is critical. Yet, so far band structure investigations of 2D TIs are scarce^4−6^ and an understanding of their dynamical properties and microscopic interactions, imperative for controlling QSH functionalities, is lacking.

A suitable approach to tackle these questions is time- and angle-resolved photoemission spectroscopy (trARPES), which has been pivotal for characterizing the electronic structure and fundamental interactions of 3D TIs, the 3D analogs of QSH insulators.^7−13^ This method grants direct access to the energy- and momentum-dependent electron dynamics after femtosecond optical excitation and to states that are not occupied in equilibrium. Thus, trARPES allows for mapping of the transiently populated states above the Fermi level, which has been essential for a clear separation of semiconducting bulk and metallic topological in-gap states in 3D TIs. Gaining a similar understanding of the electronic structure and elementary scattering processes of 2D TIs is of strong interest from both scientific and application perspectives.

A novel platform to address this knowledge gap is the room temperature QSH candidate bismuthene, that is, a monolayer of bismuth atoms arranged in a planar honeycomb geometry on a semiconducting silicon carbide SiC(0001) substrate.^14^ Spatially resolved scanning tunneling spectroscopy (STS) measurements have demonstrated a large band gap of ∼0.8 eV in bulk areas, far greater than in any other QSH system,^4−6,15−20^ and conductive 1D states at exposed sample edges near substrate terrace steps,^14,21^ as illustrated in Figure 1a. Intriguingly, in topologically nontrivial materials, additional pairs of coupled ESs can arise within the bulk areas at extended 1D defects,^22,23^ recently observed within the 2D bulk of bismuthene along structure-induced domain boundaries.^24^ However, a demonstration of the elusive ESs using a momentum-resolved probe has proven challenging, as they constitute only a marginal fraction of the total surface area. Furthermore, confirmation of the theoretically predicted large indirect bulk band gap of bismuthene and characterization of microscopic carrier scattering processes are still missing, as previous studies largely relied on momentum-integrating local probes.^14,21,24^

**Figure 1:**
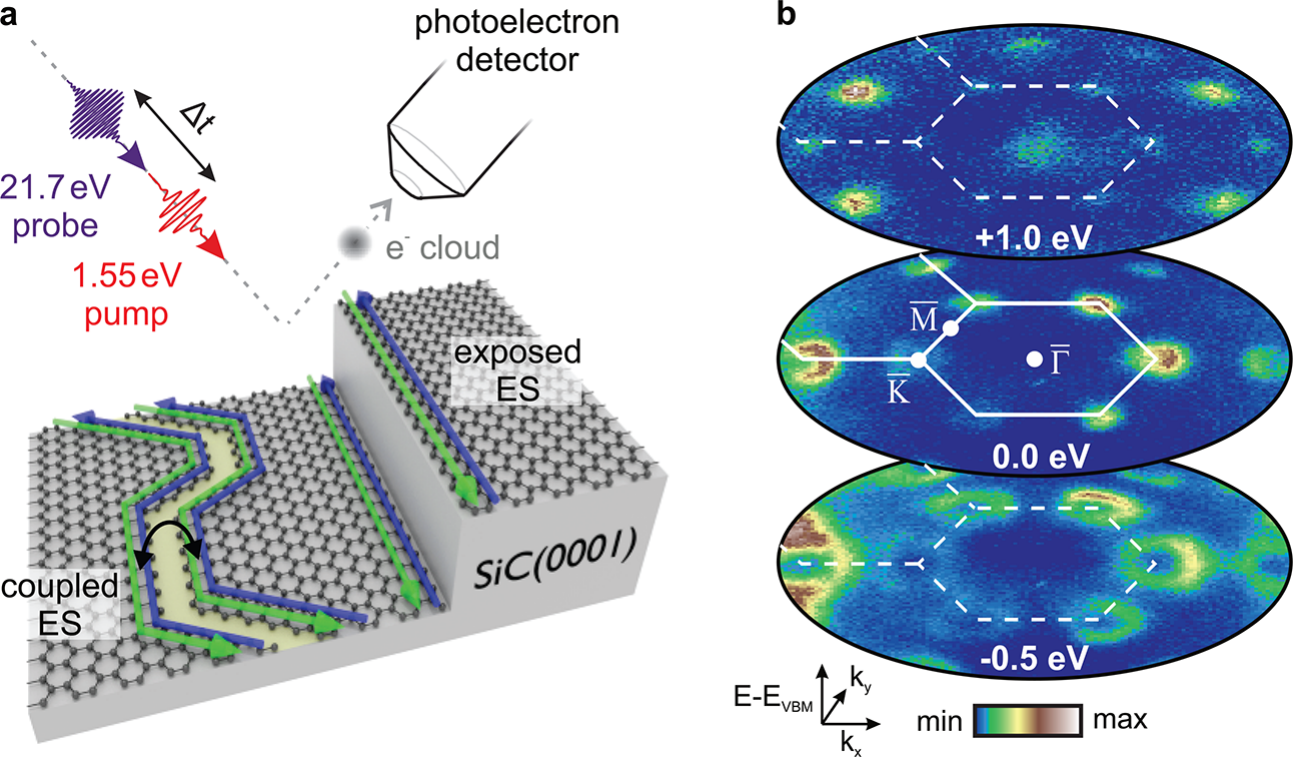
Experimental scheme and photoelectron constant-energy contours. (a) Illustration of the trARPES experiment. An optical pump pulse excites the bismuthene sample, followed by an XUV pulse that probes the electronic distribution after a time delay, Δ*t*. The green and blue arrows represent the two spin channels of the coupled ESs at a domain boundary and of the exposed ESs at a substrate step edge. (b) Constant-energy contours with radius *k*_∥_ ≈ 2 Å^–1^ of bismuthene after photoexcitation (*h*ν = 1.55 eV, Δ*t* = −75 to +75 fs). Two exemplary BZs and high-symmetry points are indicated.

Here, we investigate the ultrafast electron dynamics of photoexcited bismuthene at room temperature using trARPES, as illustrated in Figure 1a, allowing us to access the microscopic scattering channels from the dynamics of the nonequilibrium state prepared by optical excitation. Combining a hemispherical analyzer and a time-of-flight momentum microscope for photoelectron detection,^25,26^ we map the transiently populated conduction band structure and confirm the existence of a wide indirect bulk band gap of ∼0.82 eV. In addition, we identify faint gap-filling spectral weight that connects bulk valence and conduction bands, which we attribute largely to the topological ESs located at the structural domain boundaries of bismuthene.^24^ Tracking the full relaxation pathway of hot photocarriers across the entire first Brillouin zone (BZ) reveals a fast depletion of the transient conduction band population within ∼1 ps, as the in-gap states enable a highly efficient relaxation of excited carriers, incompatible with the slow recombination observed in topologically trivial indirect semiconductors.

Bismuthene was epitaxially grown on SiC(0001) substrates (see Methods). High-quality sample surfaces with low defect rates were confirmed using scanning tunneling microscopy (STM) and low-energy electron diffraction (LEED), see Supplementary Figure S1.

We begin by mapping the electronic band structure of bismuthene upon photoexcitation, as shown in Figure 1b. Strong spin–orbit coupling in combination with covalent bonding of the Bi atoms with the substrate opens a large band gap in the Dirac-like crossing at the K̅ points of the hexagonal BZ.^27^ Excitation with near-infrared optical pulses lifts charge carriers across the bulk band gap and transiently populates conduction band states localized at K̅ and, more pronounced, at the Γ̅ points of the first and second BZs 1 eV above the valence-band maximum (VBM). Next, we focus on a momentum cut along the Γ̅–K̅ direction, which features the region of the direct optical interband transition near K̅ and the conduction-band minimum (CBM) at Γ̅. Consistent with previous studies,^14^ the equilibrium band structure of bismuthene features sharp spin–orbit split low-energy valence bands at K̅ (Figure 2a). Upon optical excitation, a weak excited carrier population at K̅ and a distinct dispersive band at the CBM at Γ̅ emerge (Figures 2b,c). Concurrently, the valence bands at K̅ are depleted by the optical transition (blue colored region in Figure 2c), and their bandwidth broadens due to scattering of the photoholes with excited quasiparticles.^28,29^

**Figure 2:**
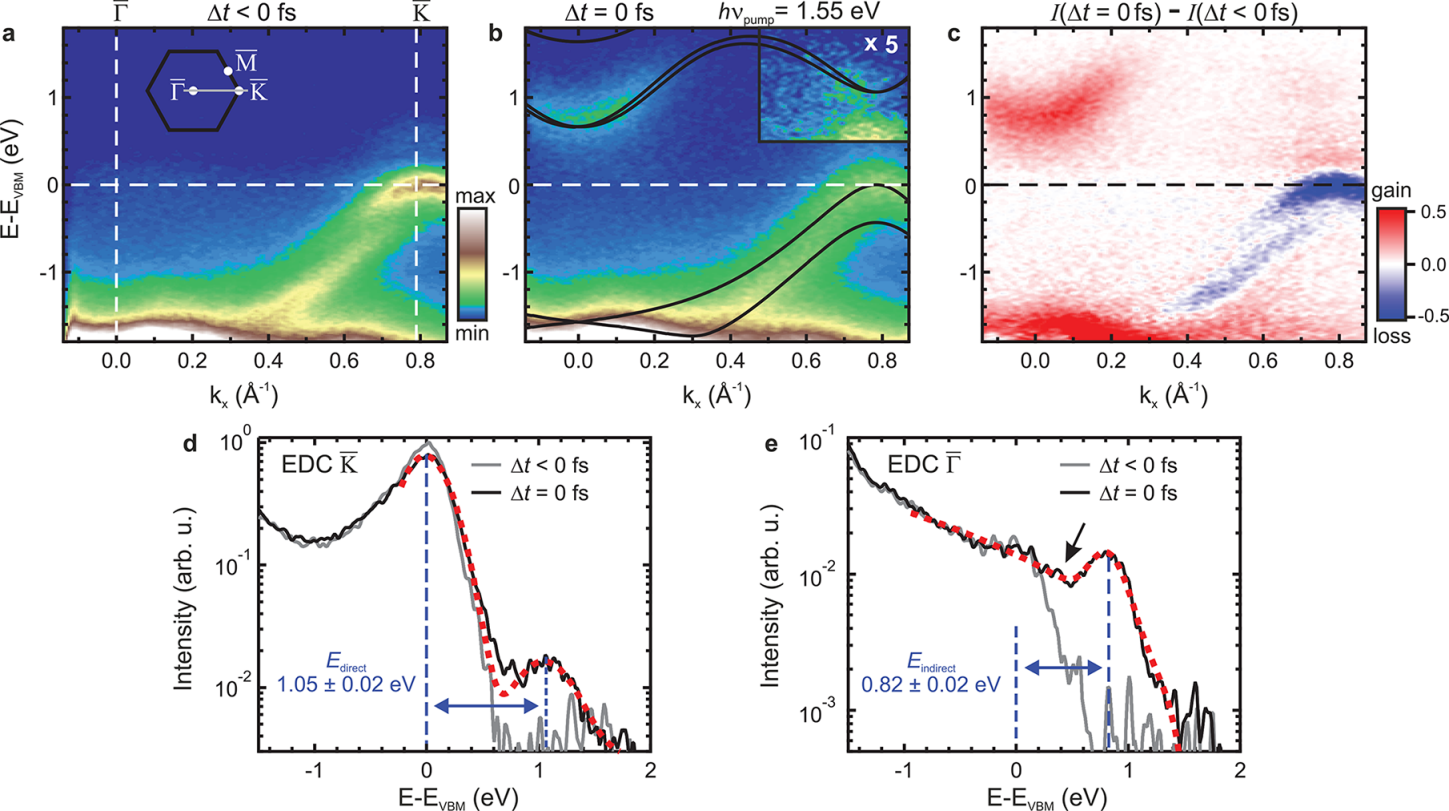
Electronic band-structure maps. (a) False-color plots of the trARPES measurements of bismuthene along the Γ̅–K̅ direction (gray line in inset) in equilibrium and (b) after optical excitation (*h*ν = 1.55 eV, incident fluence *F* = 0.50 mJ cm^–2^, Δ*t* = −40 to +40 fs). The intensity in the inset is enhanced by a factor of 5. DFT band structure calculations (black) are adopted from Reis et al.^14^ (c) Differential photoemission intensity (pre-excitation signal subtracted) at Δ*t* = 0 fs. (d, e) Energy distribution curves (EDCs) (d) at K̅ and (e) at Γ̅ in equilibrium and after weak excitation (*F* = 0.14 mJ cm^–2^, Δ*t* = −40 to +40 fs, momentum-integration ±0.05 Å^–1^). The red dashed curves mark best fits as described in the text. The black arrow indicates the in-gap intensity extending into the conduction band upon photoexcitation (see discussion). The direct and indirect band gaps are marked in blue.

Band-gap renormalization by photodoping is particularly pronounced in 2D materials due to reduced charge carrier screening.^30,31^ To minimize this effect, we extract the direct and indirect band gaps at temporal pump–probe overlap using a low incident fluence of 0.14 mJ cm^–2^, as shown in Figures 2d,e. At K̅, we find a direct band gap of 1.05 ± 0.02 eV, extracted from the peak positions of Gaussian fits to the upper spin–orbit split band at the VBM and to the lowest-lying CB, which is in excellent agreement with density functional theory (DFT) calculations (1.07 eV^14^). The indirect band gap between the VBM at K̅ and the CBM at Γ̅, extracted using a Gaussian fit with an exponentially decaying background, amounts to 0.82 ± 0.02 eV, which is in reasonable correspondence with the DFT value of 0.67 eV.^14^ Furthermore, the experimental value also perfectly agrees with the momentum-integrated bulk band gap of ∼0.8 eV obtained from STS measurements.^14^ We note that already at a low fluence of 0.14 mJ cm^–2^, the indirect quasiparticle band gap is weakly renormalized by ∼40 meV within 100 fs, resulting from the increased screening by quasi-free photocarriers (Supplementary Figure S2). For moderate fluences, we observe a significant initial band gap reduction by ∼150 meV, followed by a transient recovery, in agreement with previous studies of 2D semiconductors.^30,31^ Additional measurements using 3.1 eV optical excitation, providing a larger view into the dispersion of the lowest-energy conduction band, are shown in Supplementary Figure S3.

Next, to elucidate the quasiparticle scattering channels in the photocarrier relaxation processes of bismuthene, we investigate the excited-state population dynamics after 1.55 eV optical excitation along the Γ̅–K̅ direction (Figure 3a). As the transient photoemission intensities in Figure 3b show, a CB population builds up first near K̅ (box 1 in Figure 3a), reaching its maximum intensity at 27 fs. The apparent delay with respect to temporal pump–probe overlap is due to a buildup of the excited-state population at the K̅ valley until the end of the pump laser pulse, see the inset in Figure 3b. Subsequently, carriers appear at Γ̅ (box 2) with a delay of ∼40 fs, and last a faint intensity within the bulk band gap at Γ̅ (box 3) builds up, followed by a complete recovery on a time scale of ∼1 ps. We quantify these dynamics by employing single-exponential decay fits, characterized by the time delay at which the excited-state population reaches the maximum, *t*_max_, and the 1/*e* lifetime, τ. To establish the full energy- and momentum-dependent scattering pathway, we extend this evaluation of three exemplary areas by fitting the transient intensity of each energy–momentum bin across Figure 3a using a sliding integration window. The resulting energy–momentum maps of the fit parameters *t*_max_ and τ allow us to track the arrival time of excited carriers in energy–momentum space (Figure 3c) and provide a concise overview of the lifetimes associated with particular states (Figure 3d).

**Figure 3:**
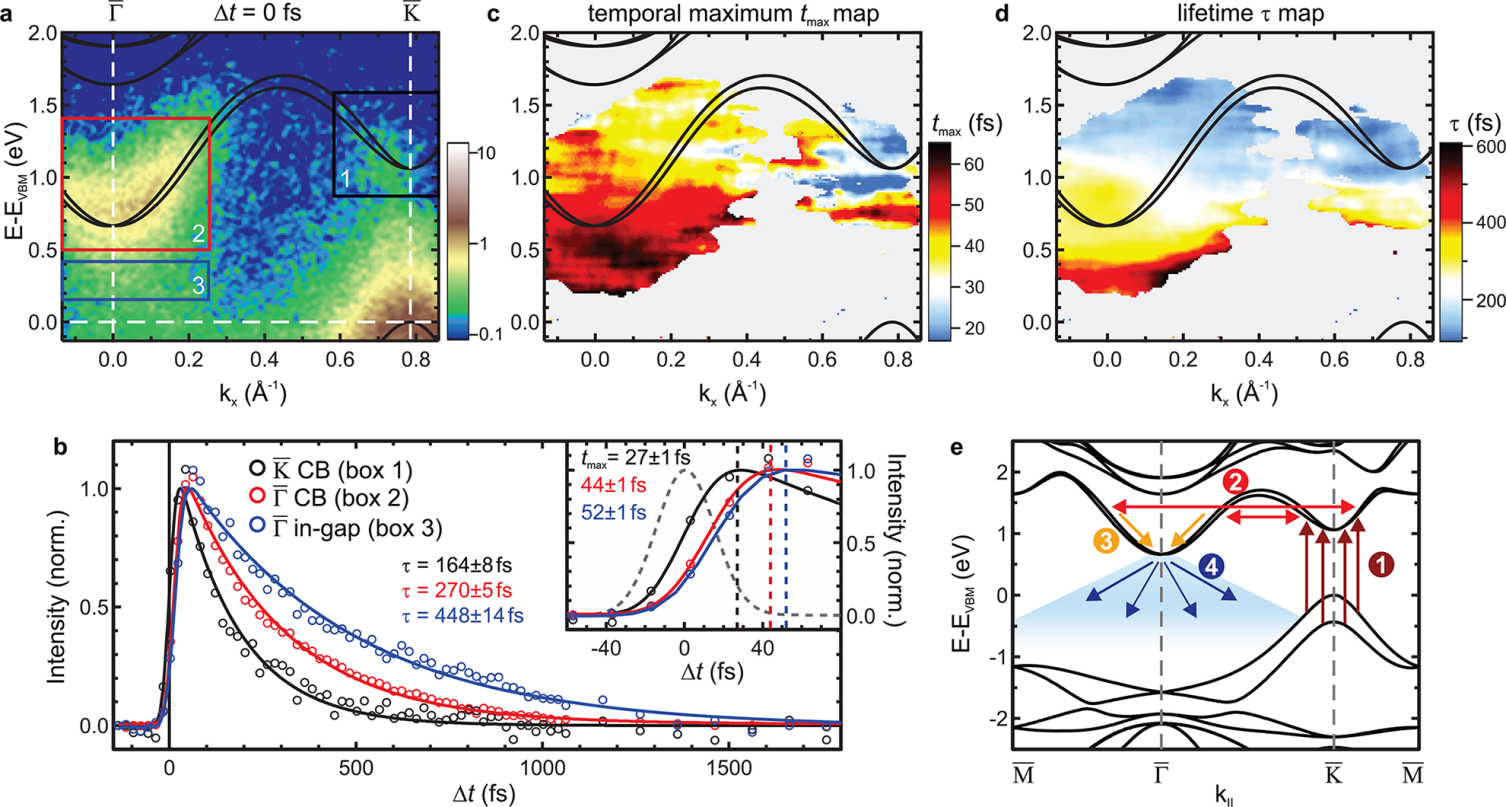
Carrier relaxation dynamics. (a) Excited-state band dispersion after 1.55 eV optical excitation (*F* = 0.50 mJ cm^–2^). (b) Normalized photoemission intensities corresponding to boxes 1–3 indicated in panel a as a function of pump–probe delay. The solid lines show best fits using a single-exponential decay convolved with a Gaussian (Gaussian width as free parameter). The fit parameters *t*_max_ (temporal intensity maximum) and τ (1/*e* decay constant) are given with one standard deviation as uncertainty. Inset: dynamics near Δ*t* = 0 fs. The gray dashed line indicates the temporal profile of the pump-laser pulse. (c) Temporal maximum *t*_max_ and (d) carrier lifetimes τ from bin-wise energy- and momentum-dependent decay fits. For this, the transient photoemission intensities are extracted across the energy-momentum region shown in panel a using a sliding-window integration (Δ*E* = 0.1 eV, Δ*k*_*x*_ = 0.15 Å^–1^) and fitted using the function described above. Regions with low photoemission intensity or large fit uncertainties (σ_*t*_max__ > 10 fs, σ_τ_ > 40 fs) are masked in gray. (e) Schematic scattering processes within the DFT band structure (see text). The in-gap states are indicated in blue.

Combining the results of both maps yields a detailed picture of the complete carrier relaxation pathway, schematically depicted in Figure 3e: (1) Carriers are initially injected by a vertical interband transition into the CB near K̅ using 1.55 eV radiation. (2) The hot electrons redistribute by intervalley scattering, which spreads the carriers over an extended momentum region into the Γ̅ valley on a 10 fs time scale, a phenomenon commonly observed in photoexcited semiconductors.^32−35^ (3) Subsequently, hot carriers relax toward the CBM at Γ̅ via electron–electron and electron–phonon scattering within ∼50 fs. Although bulk bismuthene exhibits an indirect band gap of nearly 1 eV, the lifetime of the conduction band population is only on the order of few 100 fs, an orders of magnitude faster relaxation than in conventional indirect semiconductors.^32,36−39^ (4) These ultrashort lifetimes indicate a highly efficient carrier relaxation. The question naturally arises which states other than the insulating 2D bulk states in bismuthene could mediate the observed fast decay. Intriguingly, we observe faint gap-filling spectral weight reaching up to the CBM for several 100 fs after photoexcitation (Supplementary Figure S4), which we discuss below. A relaxation of the conduction band population through these in-gap states is supported by the fact that they are populated last and feature the longest lifetimes. Finally, within ∼1.5 ps, also the in-gap states above the VBM are fully depleted. Note that the extracted population lifetimes are distinct from single-particle lifetimes that are directly encoded in the electron self-energy, and thus only represent an upper limit for the time scale of scattering processes.^40,41^

Lastly, we examine the in-gap spectral weight, and we discuss its origin. We find that already in equilibrium a faint intensity islocated at Γ̅ reaching up to *E*_VBM_ (Figure 4a). Upon optical excitation, the in-gap intensity extends into the CBM (Figure 4b), resulting in the absence of an explicit gap between bulk conduction and valence bands (black arrow in Figure 2e). While it may seem obvious to assign the in-gap feature to the topological ESs that were directly probed in previous STM/STS studies,^14,21,24^ a careful examination is required.

**Figure 4:**
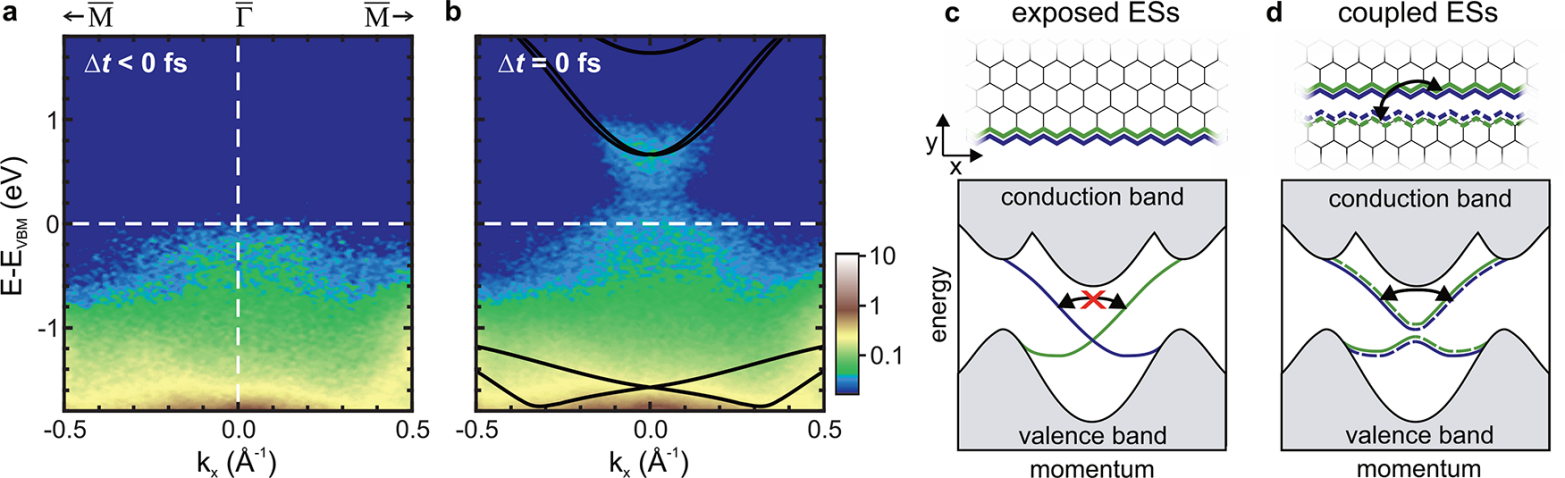
In-gap intensity. (a) Photoemission spectra at Γ̅ in equilibrium and (b) after optical excitation (*h*ν = 1.55 eV, *F* = 0.32 mJ cm^–2^). Faint spectral weight is located between the band gap predicted by bulk DFT calculations (black), connecting the valence and conduction states. Visibility of the in-gap states is enhanced by a logarithmic color scale. (c, d) (top) Sketch of helical ESs (blue, green) at an exposed sample edge with zigzag termination and of coupled ESs at a domain boundary, respectively. (bottom) Schematic dispersion of infinitely extended exposed and coupled 1D ESs, respectively. The projected bulk band structure is indicated in gray. The hybridization of coupled ES pairs opens an energy gap and lifts spin-momentum locking, enabling single-particle backscattering, indicated by the black arrow. The size of the gap opening at the crossing of the ES dispersion, however, is expected to be significantly lower than our experimental energy resolution of ∼150 meV. Adopted from refs (14 and 24).

For that, we compare the relaxation dynamics and the in-gap feature for bismuthene grown on two different substrate types, that is, intentionally miscut and planar SiC substrates (Supplementary Figure S1). Using a miscut substrate yields a high density of unidirectional, topologically protected exposed ES along the parallel substrate step edges, as illustrated in Figure 4c, while bismuthene prepared on a planar substrate features only a negligible density of (randomly oriented) substrate steps with exposed ESs. Yet, for both substrate types, additional topological ESs arise at structural phase-slip domain boundaries within the 2D bulk of bismuthene. These domain boundaries result from the fact that the  Bi honeycombs can have three distinct registries with respect to the substrate lattice, causing the growth of domains. Pairs of helical ESs emerge at the zigzag edges on either side of these boundaries, see Figure 4d, lifting the topological protection through mutual hybridization and leading to a mixing of different helicities, as confirmed by local tunneling spectroscopy.^24^

The presented characterization of the in-gap feature and relaxation dynamics was conducted on bismuthene prepared on a miscut substrate. However, in bismuthene prepared on a planar substrate featuring only a negligible density of exposed ESs, equivalent relaxation dynamics (Supplementary Figure S5) and a similar in-gap feature (Supplementary Figures S4 and S6) are observed. This leads us to the conclusion that the in-gap intensity primarily originates from topological ESs that arise at structural domain boundaries, which are present in both planar and miscut samples. The faint intensity of the in-gap states on the order of a few percent of the bismuthene bulk bands at K̅ is consistent with the assignment to coupled ESs, as the domain boundaries constitute only a fraction of the probed surface. We further conclude that the relaxation of excited-state charge carriers must be strongly facilitated by the quasi-metallic density of states observed at such domain boundaries, enabling the rapid depletion of the conduction band population, analogous to the depletion of conduction band populations by topological surface states in photoexcited 3D TIs.^8^ Since we do not observe large deviations for the decay times in the case of the bismuthene sample grown on a miscut substrate (Supplementary Figure S5), any definite conclusion on the additional role of the exposed topological ESs is difficult to draw at this point. Future developments in (time-resolved) nano-ARPES featuring a nanometer spatial resolution may allow isolation of the spectral features of exposed ESs at substrate terrace steps and individual pairs of coupled ESs at domain boundaries.

While the coupled ESs at domain boundaries consistently explain the in-gap spectral weight and short photocarrier lifetimes, several alternative scenarios can potentially induce a continuous in-gap intensity. First, impurities and defects may provide additional states within the bulk band gap. However, STM/STS studies show that only domain boundaries and exposed sample edges host in-gap states, while all other sample regions remain fully gapped.^14,21,24^ In addition, the observed confinement of the in-gap intensity to the momentum-region near Γ̅ speaks against impurities as primary origin, as such defect states typically lack a clear momentum dependence.^42^ Second, a large energy line-width of the bismuthene bulk valence band may lead to a metal-like extension of intensity up to the Fermi level. However, analogous to the first line of argument, spatially resolved STS measurements unambiguously demonstrate insulating behavior in bulk regions,^14^ indicating that the observed continuous in-gap intensity is not connected to bulk bismuthene but rather to ESs. Thus, by excluding alternative interpretations and consistent with earlier local tunneling spectroscopy results,^24^ we assign the in-gap spectral weight largely to coupled ESs at domain boundaries. As ESs forming along extended 1D defects critically limit the lifetime of excited carriers and may also pose challenges for applications utilizing the spin-selective transport along exposed sample edges,^23,43^ our study underlines the need for high-quality sample surfaces.

In conclusion, we experimentally mapped the electronic band structure of the quantum spin Hall insulator bismuthene after near-infrared photoexcitation and determined the direct and indirect band gaps of ∼1.1 eV and ∼0.8 eV, respectively. Analysis of the microscopic scattering pathway of hot photocarriers revealed exceptionally fast carrier relaxation dynamics governed by faint in-gap states located within the indirect band gap, which correspond with the topological edge states arising at bismuthene domain boundaries. The demonstration of a large fundamental band gap and the in-gap spectral weight persisting at room temperature and under strong optical excitation highlight the promising role of bismuthene as an ambient-condition quantum spin Hall candidate. Additionally, due to the exceptionally large band gap, bismuthene serves as a unique platform for optically addressing novel functionalities based on the topological edge states and for studying excitons in a topologically nontrivial system. Our insights gained on quasiparticle scattering processes lay the basis for future studies of sub-band gap excitations and optical control schemes of edge-state currents.^44,45^

## Methods

### Sample Preparation and STM Measurements

Bismuthene was epitaxially grown on n-doped 4H-SiC(0001) substrates (0.01–0.03 Ω·cm, carrier concentration ∼10^18^–10^19^ cm^–3^, planar and 4° miscut) in ultrahigh vacuum <10^–10^ mbar. Prior to growth, a smooth H-terminated SiC surface was prepared by hydrogen-based dry-etching. Growth was performed at ∼600 °C to thermally desorb the surface H-termination, while simultaneously offering Bi atoms from a commercial effusion cell.^14^ Successful growth of low-defect bismuthene samples was verified using low-energy electron diffraction and scanning tunneling microscopy.

### Time-Resolved ARPES Measurements

After characterization, the samples were transferred to the trARPES setup using an UHV suitcase at *p* < 10^–10^ mbar. All measurements were performed at room temperature using a laser-based high-harmonic-generation trARPES setup (p-polarized probe at *h*ν_probe_ = 21.7 eV, s-polarized pump at *h*ν_pump_ = 1.55, 3.10 eV, 500 kHz repetition rate, Δ*E* ≈ 150 meV, Δ*t* ≈ 40 fs) with a 6-axis manipulator (SPECS Carving).^25^ Photoelectrons were detected with either a hemispherical analyzer (SPECS Phoibos 150) or a time-of-flight momentum microscope (SPECS METIS 1000).^26^ The momentum microscope allows for parallel acquisition of the 3D photoelectron distribution *I*(*E*_kin_, *k*_*x*_, *k*_*y*_) across a large energy and momentum range and was thus utilized for overview measurements of the electronic band structure (Figure 1). In contrast, the hemispherical analyzer allows for fast data acquisition within a limited energy-momentum window, and was thus used to map selected high-symmetry directions (Figures 2–4). The data presented in Figure 1 were acquired on a planar substrate and that in Figures 2–4 on a miscut substrate. The extreme ultraviolet (XUV) probe spot size (fwhm) was ∼80 × 80 μm^2^. The pump spot sizes were ∼260 × 200 μm^2^ (*h*ν = 1.55 eV) and ∼510 × 475 μm^2^ (*h*ν = 3.10 eV). All fluences stated in the text correspond to incident fluences. Temporal pump–probe overlap was determined from the pump-laser-induced depletion of the valence band population, as shown in Supplementary Figure S7.

## Supporting Information Available

STM and LEED surface characterization of the bismuthene samples, characterization of the time-dependent quasiparticle band gap, mapping of the conduction-band dispersion after 3 eV optical excitation, additional trARPES characterization of the in-gap spectral weight, and determination of temporal pump–probe overlap (PDF)

## Notes

All data that support the findings of this study are publicly available at the Zenodo data repository, 10.5281/zenodo.5512069.
