# Supplementary material for: Ultrafast Momentum-Resolved Hot Electron Dynamics in the Two-Dimensional Topological Insulator Bismuthene
Source: Nano Lett. 2022 Jun 16;22(13):5420–6. doi: 10.1021/acs.nanolett.2c01462 (PMC9284614; doi:10.1021/acs.nanolett.2c01462)
Supplement: Supplementary file 1 — nl2c01462_si_001.pdf [file nl2c01462_si_001.pdf]

Supporting Information for "Ultrafast Momentum-resolved Hot Electron Dynamics in the Two-dimensional Topological Insulator Bismuthene"

Julian Maklar[1], Raúl Stühler[2], Maciej Dendzik[1,3], Tommaso Pincelli[1], Shuo Dong[1], Samuel Beaulieu[1,4], Alexander Neef[1], Gang Li[5], Martin Wolf[1], Ralph Ernstorfer[1,6], Ralph Claessen[2], and Laurenz Rettig[1]

[1] *Fritz-Haber-Institut der Max-Planck-Gesellschaft, Faradayweg 4-6, D-14195 Berlin, Germany*
[2] *Physikalisches Institut and Würzburg-Dresden Cluster of Excellence ct.qmat, University of Würzburg, D-97070 Würzburg, Germany*
[3] *Current address: Department of Applied Physics, KTH Royal Institute of Technology, Hannes Alfvéns väg 12, 114 19 Stockholm, Sweden*
[4] *Current address: Université de Bordeaux - CNRS - CEA, CELIA, UMR5107, F33405, Talence, France*
[5] *School of Physical Science and Technology, ShanghaiTech University, Shanghai 200031, China*
[6] *Institut für Optik und Atomare Physik, Technische Universität Berlin, Straße des 17. Juni 135, 10623 Berlin, Germany*

June 11, 2022

Supplementary Figures

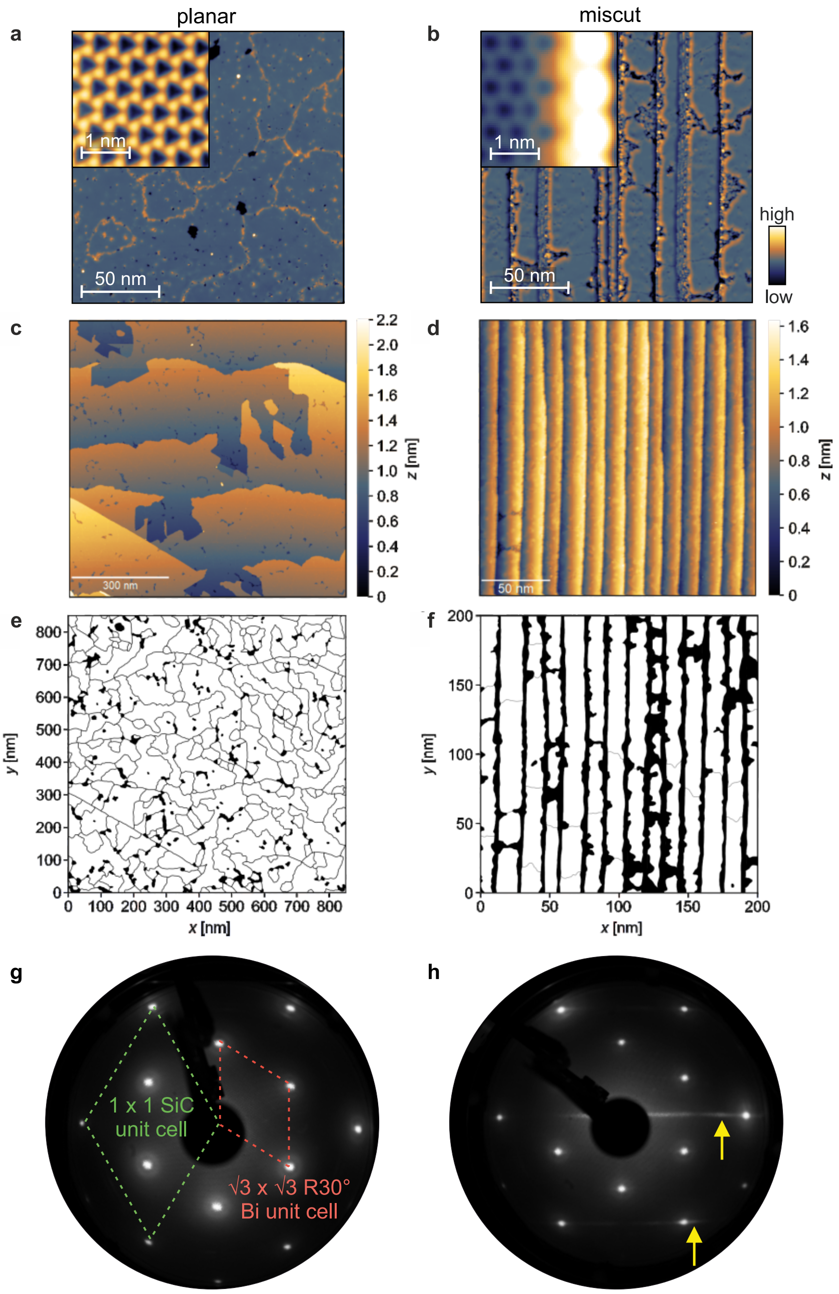

Supplementary Fig. S1: Surface characterization of bismuthene samples. (a) STM constant-current image of bismuthene on a planar SiC substrate. A meandering network of domain boundary segments (bright ridges) intersects the bismuthene film into connected domains. Inset: Close-up of the Bi honeycomb lattice. (b) STM image of bismuthene on a 4° miscut substrate featuring unidirectional SiC terrace steps every $\sim$ 15 nm that induce exposed bismuthene edges. Inset: Bi honeycombs near a step edge. While both the morphology of the planar and the miscut bismuthene samples feature domain boundaries, the miscut bismuthene sample exhibits a significantly larger exposed edge density. Scan parameters: $V_{\mathrm{set}} = 3.0\,\mathrm{V}$, $I_{\mathrm{set}} = 50\,\mathrm{pA}$, $T = 4.35\,\mathrm{K}$; insets: $V_{\mathrm{set}} = -0.8\,\mathrm{V}$, $I_{\mathrm{set}} = 100\,\mathrm{pA}$, $T = 4.35\,\mathrm{K}$. (c and d) Overview STM constant current images of bismuthene on a planar and a miscut substrate, respectively. Scan parameters: (c) $V_{\mathrm{set}} = 3.0\,\mathrm{V}$, $I_{\mathrm{set}} = 50\,\mathrm{pA}$, $T = 4.35\,\mathrm{K}$; (d) $V_{\mathrm{set}} = 2.6\,\mathrm{V}$, $I_{\mathrm{set}} = 30\,\mathrm{pA}$, $T = 4.35\,\mathrm{K}$. (e and f) Binary masks marking domain boundaries and defective areas on the planar and miscut samples from (c) and (d), respectively. (g) Low-energy electron diffraction of bismuthene on a planar SiC substrate recorded at an energy of 48 eV and (h) on a miscut substrate at 50 eV. Sharp, intense diffraction spots and a weak diffuse background signal indicate high-quality sample surfaces. The yellow arrows in (h) mark stripe-like elongations of the SiC spots corresponding to unidirectional substrate step edges. Exemplary SiC 1x1 and Bi $\sqrt{3}$x$\sqrt{3}$ R30° reciprocal unit cells are indicated.

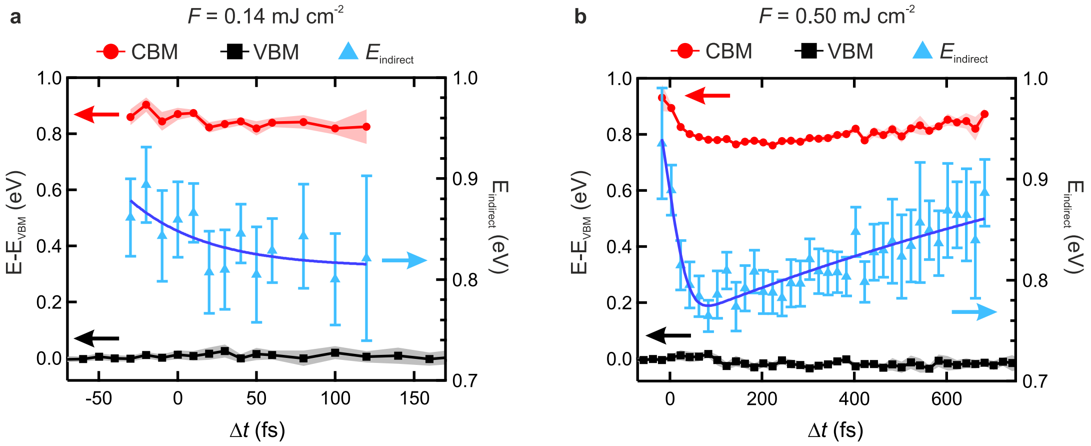

Supplementary Fig. S2: Renormalization of the quasiparticle band gap. Position of the CBM and VBM (left axis) and extracted indirect band gap (CBM-VBM difference, right axis) as a function of pump-probe delay for (a) low and (b) moderate incident fluence. When applying a low fluence of $F = 0.14\,\mathrm{mJ\,cm^{-2}}$ (panel a), the VBM position remains approximately constant while the CBM position shifts slightly downwards within 100 fs, resulting in a reduction of the indirect band gap by $\sim$ 40 meV. However, in this fluence regime, the limited number of hot carriers in combination with their fast relaxation allows for reliable tracking of the CBM only up to $\sim$ 120 fs. For a moderate fluence of $F = 0.50\,\mathrm{mJ\,cm^{-2}}$ (panel b), we observe a pronounced time-dependent band-gap renormalization, as the CBM undergoes a significant shift while also the position of the VBM changes with Δt. The photoexcited quasi-free carriers initially increase the screening of the Coulomb interactions, transiently reducing the effective band-gap size by $\sim$ 150 meV. As the system relaxes to equilibrium, the band gap recovers with increasing Δt. The solid blue lines in (a) and (b) serve as guides to the eye. The band positions were extracted using Gaussian fits as discussed in the main text. The error bands and bars correspond to one standard deviation resulting from the Gaussian fits.

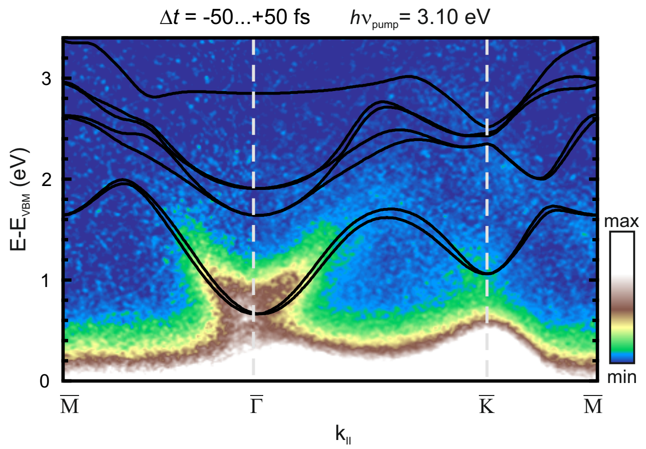

Supplementary Fig. S3: Conduction band dispersion after photoexcitation. Photoemission intensity along the high-symmetry momentum directions after 3.1 eV optical excitation (F=0.03 mJ cm^{-2}) at temporal pump-probe overlap. DFT calculations are shown in black.

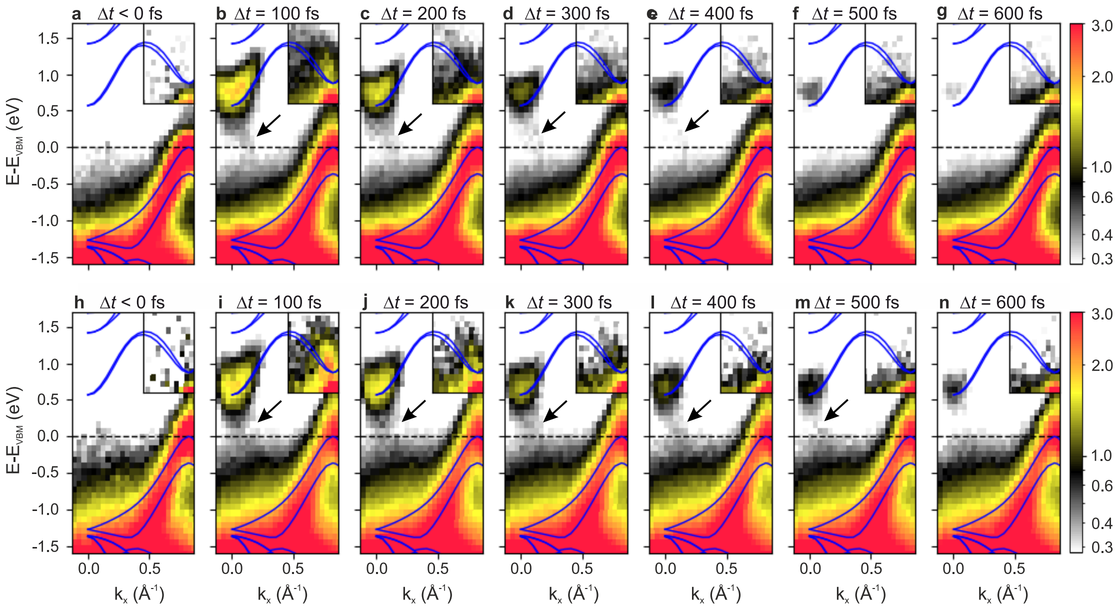

Supplementary Fig. S4: Dynamic band-structure maps. (a-g) False-colour plots of the trARPES measurements of bismuthene on a miscut substrate along the $\overline{\Gamma}$-$\overline{\mathrm{K}}$ direction for selected time delays (time integration window of 200 fs, $h\nu = 1.55\,\mathrm{eV}$, F=0.50 mJ cm^{-2}). (h-n) Equivalent measurements for bismuthene on a planar substrate. The black arrows indicate the in-gap intensity near $\overline{\Gamma}$. The intensity in the insets at $\overline{\mathrm{K}}$ is enhanced. DFT band structure calculations are shown in blue.

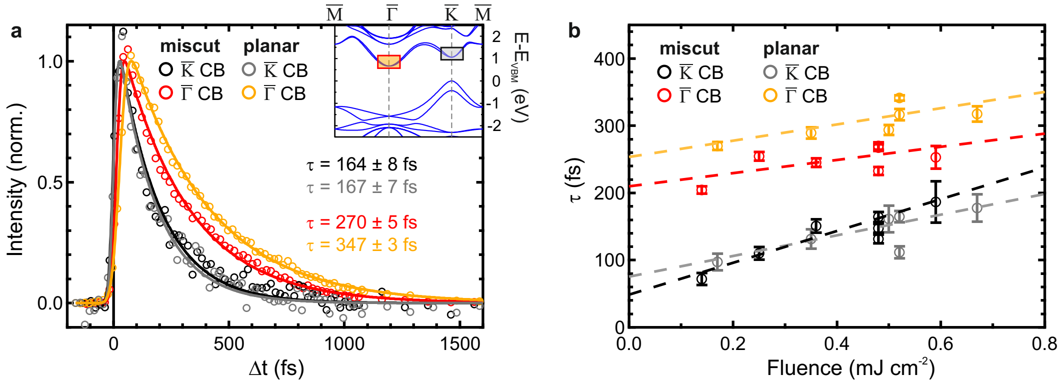

Supplementary Fig. S5: Photocarrier lifetimes. (a) Normalized photoemission intensities of the conduction band populations at $\overline{\mathrm{K}}$ and $\overline{\Gamma}$ for bismuthene on miscut and planar substrates (F=0.50 mJ cm^{-2}) versus pump-probe delay. The inset indicates the energy-momentum regions of interest within the DFT band structure of the respective time traces (equivalent to boxes 1 and 2 in Fig. 3a). Single-exponential decay fits reveal similar $1/e$ lifetimes of the populations at $\overline{\mathrm{K}}$ for both substrate types, and slightly increased lifetimes at $\overline{\Gamma}$ for the planar substrate. (b) Extracted lifetimes for both substrate types as function of incident fluence. The dashed lines serve as guides to the eye. While the overall lifetime increases with fluence, the lifetime at $\overline{\Gamma}$ for the planar substrate is systematically higher with respect to the miscut substrate for all applied fluences. The error bars correspond to one standard deviation of the fit parameter τ.

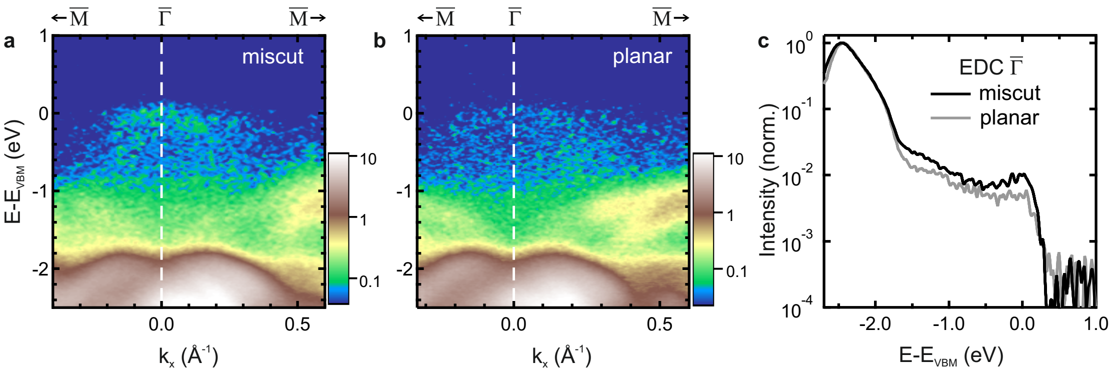

Supplementary Fig. S6: In-gap feature at $\overline{\Gamma}$. (a) Equilibrium band dispersion of bismuthene on a miscut substrate. A faint feature near the VBM is identified, which we assign to topological ESs. (b) Band dispersion of bismuthene on a planar substrate, exhibiting a similar, slightly less pronounced feature at $\overline{\Gamma}$. (c) Corresponding EDCs at $\overline{\Gamma}$. Both samples show a faint but distinct intensity up to E_{VBM}, which is roughly a factor 2 more intense for the miscut substrate.

Determination of temporal pump-probe overlap

To determine the temporal pump-probe overlap $\Delta t = 0\,\mathrm{fs}$, we extract the initial depletion of the valence band population at $\overline{\mathrm{K}}$ (red box in Supplementary Fig. S7a) resulting from the vertical optical transition. The extracted photoemission intensity as function of pump-probe delay is fitted using an error-function, see Supplementary Fig. S7b. Here, the central position of the error function corresponds to the temporal peak of the optical pump pulse, t_0. For all measurements presented in this manuscript, we calibrate the pump-probe delay such that $\Delta t = 0\,\mathrm{fs}$ corresponds to t_0.

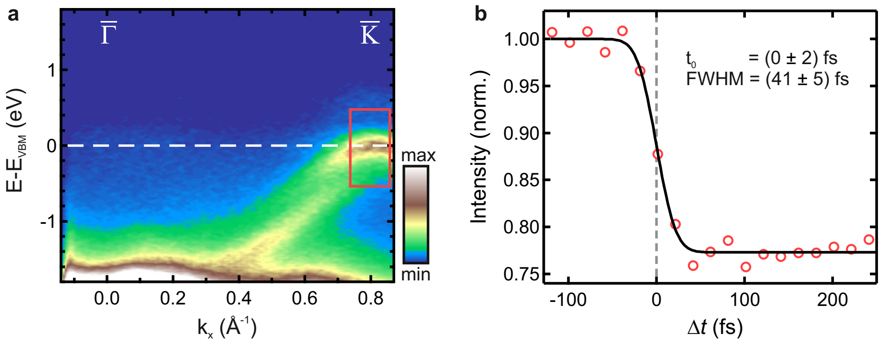

Supplementary Fig. S7: Arrival time of the optical excitation pulse. (a) Equilibrium ARPES measurement of the $\overline{\Gamma}$-$\overline{\mathrm{K}}$ direction. (b) Normalized photoemission intensity extracted from the red box in panel (a) as function of pump-probe delay (red circles, $h\nu_{\mathrm{pump}} = 1.55\,\mathrm{eV}$, incident fluence F=0.50 mJ cm^{-2}). The black solid line marks the best fit using an error function. Fit coefficient values of the temporal peak t_0 and full width at half maximum of the pump pulse are stated in the figure, respectively. One standard deviation is given as uncertainty.
